# Draft Genome Sequence of Citrobacter gillenii MBT-C3, Isolated from Lamb’s Lettuce

**DOI:** 10.1128/MRA.01177-18

**Published:** 2018-10-18

**Authors:** Gyu-Sung Cho, Maria Stein, Wilhelm Bockelmann, Horst Neve, Erik Brinks, Charles M. A. P. Franz

**Affiliations:** a Department of Microbiology and Biotechnology, Max Rubner-Institut, Federal Research Institute of Nutrition and Food, Kiel, Germany; University of Rochester School of Medicine and Dentistry

## Abstract

The genome of the streptomycin-resistant Citrobacter gillenii strain MBT-C3, isolated from lamb’s lettuce in Germany, was sequenced. Sequence analysis showed the assembled draft genome size to be 5,167,205 bp, containing a predicted total of 5,011 protein-encoding genes, 8 rRNAs, and 71 tRNAs.

## ANNOUNCEMENT

Citrobacter spp. are opportunistic human pathogens which can cause serious diseases in neonates, children, and immunocompromised patients (1, 2). Citrobacter spp. occur in the soil, in water, in food, and in the gastrointestinal tract in humans and animals and have been reported to cause diseases, such as sepsis and respiratory and urinary tract infections (2). The microbiological and medical importance of these bacteria stems from not only their pathogenicity but also the appearance of strains with multiple resistance to antibiotics used in therapy.

Currently, the genus Citrobacter comprises 15 species, of which C. gillenii was first described by Brenner et al. (3). Before that time, this species was known as Citrobacter genomospecies 10, and strains of this species were isolated from human and animal stool, human urine, and human blood (4). The most recently described Citrobacter species is C. portucalensis, which was isolated from a water well sample collected in Cantanhede, a city in the Centro Region of Portugal, in 2017 (5). We recently reported the draft genome sequence of a multidrug-resistant C. portucalensis strain isolated from vegetables (Piper guineense, also known as Ashanti pepper or uziza) in Africa (6). In this study, we sequenced the genome of a novel streptomycin-resistant C. gillenii strain, MBT-C3, isolated from lamb’s lettuce grown in Germany.

The Citrobacter gillenii MBT-C3 strain was grown overnight in brain heart infusion (BHI) broth, and the genomic DNA was extracted using the ZR fungal/bacterial DNA miniprep kit (Zymo Research, Freiburg, Germany) and quantified using a Qubit 3 fluorometer (Invitrogen, Darmstadt, Germany). For sequencing, the Nextera XT DNA library preparation kit and the MiSeq reagent kit version 2 were used, according to the manufacturers’ instructions, using a MiSeq sequencer (Illumina, Munich, Germany). Raw sequencing data were quality trimmed using Trimmomatic (7); the 401,324 filtered paired-end reads (2 × 250 bp) and 46,088 filtered unpaired-end reads (250 bp) were *de novo* assembled into 37 contigs, with a total length of 5,167,205 bp and a G+C content of 52.52 mol%, using SPAdes version 3.12.0 (8). The assembled genome showed 24-fold coverage, and the *N*_50_ value was 413,666 bp. The genome annotation was performed automatically with RAST (9), and a manual comparison of predicted open reading frames (ORFs) with proteins was carried out using PATRIC (10). The genome sequence annotation showed 5,011 predicted genes, 71 tRNA, 6 5S rRNA, 1 16S rRNA, and 1 23S rRNA gene. None of the contigs were identified as a plasmid-related sequence by the PlasmidFinder pipeline (11). In addition, *in silico* DNA-DNA hybridization was carried out using the Genome-to-Genome Distance Calculator (GGDC) (12). The results showed that the C. gillenii MBT-C3 genome was distinct from the genomes of representative strains of related species (i.e., Citrobacter braakii CIP 104554^T^, Citrobacter werkmanii BF6^T^, C. freundii ATCC 8090^T^, and C. portucalensis A60^T^), with similarity values of 31.6%, 31.5%, 31.4%, and 31.7%, respectively.

### Data availability.

The whole-genome shotgun project of C. gillenii MBT-C3 has been deposited at DDBJ/ENA/GenBank under the accession no. QVEK00000000 (BioProject no. PRJNA485440).
